# Supplementary material for: Interlocking of co-opted developmental gene networks in Drosophila and the evolution of pre-adaptive novelty
Source: Nat Commun. 2023 Sep 15;14:5730. doi: 10.1038/s41467-023-41414-3 (PMC10504328; doi:10.1038/s41467-023-41414-3)
Supplement: Supplementary file 1 — Supplementary information [file 41467_2023_41414_MOESM1_ESM.pdf]

Supplemental information

Interlocking of co-opted developmental gene networks in *Drosophila* and the evolution of pre-adaptive novelty

Sara Molina-Gil[1,2], Sol Sotillos[1], José Manuel Espinosa-Vázquez[1,3], Isabel Almudi[1,4] and James C-G Hombría[1*]

Supplementary Table 1: Oligonucleotides used for constructs, probes and EMSA assays.

	Oligo	5' – 3' Sequence
CRISPR	enD gRNA1	GGGACCACACACGACGATCGAGG
	enD gRNA2	GCGCTGATTCCTCCGATTAGGGG
enD constructs	enD 1 for	CATCAGGCTGCTCAATGGAC
	enD 2 for	TCTTCATACTCTGCCTGAACAA
	enD 3 rev	CTCGATACCTGAGATCCCGT
	enD 4 rev	GCGTTTCTTTCGTACCCTGG
	en DEOL for	AGATCGATTTCCCCACATGACTCGCC
	en DEOL rev1.2	GCTGTGTAAAAATAAACATTGTTCGCAC
enD0.4 mutagenesis	enD04 mut359 for	GCTCAGTTCGCAGCACTCGGATccgTGGCCAACAG
	enD04 mut359 rev	TGTTGGCCAcggATCCGAGTGCTGCGAACTGAG
	enD04 mut260 for	GGAGTTGGGCCCAcggAAATCACATCAAAGTCGC
	enD04 mut260 rev	GCGACTTTGATGTGATTTccgTGGGCCCAACTCC
	enDdwn Stat-mut for	GGCTATATCTttccagcgttTTGGATGTCTTTCACC
	enDdwn Stat-mut rev	AGACATCCAAaacgctggaaAGATATAGCCTGCCC
in situ Probe	T7 rev	AGGGATCCTAATACGACTCACTATAGGGCCCGGG GC
	EN-probe FOR	CCAAGATGAGCGATGCCAAT
	EN-probe REV	CCCGGGGCCCACATCTCGTTCTTGCCG
Upd 0.43	upd F2 M for	ATGGTCTCGTTCTTGTGAAG
	upd F2 AT rev	CCCAGGCGGGTCCAAAAATAAC
Sal 2.1	Sal 2.1 For	TCACCCTTAATGACATCTTATCAATCTGG
	Sal 2.1 Rev	TCTTATTATTAGGAATCCC

EMSA WT oligos	1F 1-78	ggAGATCGATTTCCCCACATGACTCGCCCATTTAATTGTTTTGATCTCAATTAAAGCAATTTGCCTATCGTTCACGAATA
	1R	ggTATTCGTGAACGATAGGCAAATTGCTTTAATTGAGATCAAAACAATTAAATGGGCGAGTCATGTGGGGAAATCGATCT
	2F 70-148	ggCACGAATAAGGGCAGGCTATATCTttccagcgaaTTGGATGTCTTTCACCATTAATTTGACGCGGAGATCAGCTCAAT
	2R	ggATTGAGCTGATCTCCGCGTCAAATTAATGGTGAAAGACATCCAAttcgctggaaAGATATAGCCTGCCCTTATTCGTG
	3F 140-218	ggAGCTCAATTACACCACTTCAGTCTTTTATCGCACTAATTACACTGGAACACTGGCACGGCCGGGATTTGCTGGGATTG
	3R	ggCAATCCCAGCAAATCCCGGCCGTGCCAGTGTTCCAGTGTAATTAGTGCGATAAAAGACTGAAGTGGTGTAATTGAGCT
	4F 210-288	ggTGGGATTGCTAGCGACTCGGGGATTTAGGGTCTTTGGAGTTGGGCCCATAAAAATCACATCAAAGTCGCGTCCGTTTC
	4R	ggGAAACGGACGCGACTTTGATGTGATTTTTATGGGCCCAACTCCAAAGACCCTAAATCCCCGAGTCGCTAGCAATCCCA
	5F 280-358	ggTCCGTTTCGAAAACTAGTTGCTATAAAACTGCAATTACCCGGCATGCACTAAAACGCTCAGTTCGCAGCACTCGGATT
	5R	ggAATCCGAGTGCTGCGAACTGAGCGTTTTAGTGCATGCCGGGTAATTGCAGTTTTATAGCAACTAGTTTTCGAAACGGA
	6F 358-430	ggCTCGGATTTATGGCCAACAGAAAAAGGCATTTAAGGAACAACGAAAAAGAAAAATAGAGAATGGTGGGGCCAGGGTAC
	6R	ggGTACCCTGGCCCCACCATTCTCTATTTTTCTTTTTCGTTGTTCCTTAAATGCCTTTTTCTGTTGGCCATAAATCCGAG

EMSA Short oligos and mutant versions	2 85-117 for	ggGGCTATATCTttccagcgaaTTGGATGTCTTTC
	2 85-117 rev	ggGAAAGACATCCAAttcgctggaaAGATATAGCC
	2 85-117 mutST for	ggGGCTATATCTttccagcgttTTGGATGTCTTTC
	2 85-117 mutST rev	ggGAAAGACATCCAAaacgctggaaAGATATAGCC
	4 246-284 for	ggGGAGTTGGGCCCATAAAAATCACATCAAAGTCGCGTCCG
	4 246-284 rev	ggCGGACGCGACTTTGATGTGATTTTTATGGGCCCAACTCC
	4 246-284 mut260 for	ggGGAGTTGGGCCCAcggAAATCACATCAAAGTCGCGTCCG
	4 246-284 mut260 rev	ggCGGACGCGACTTTGATGTGATTTccgTGGGCCCAACTCC
	4 246-284 mut-sal for	ggGGAGTTGGGCCCATAAAAcgCACcgCAcgGTCGCGTCCG
	4 246-284 mut-sal rev	ggCGGACGCGACcgTGcgGTGcgTTTTATGGGCCCAACTCC
	4 246-284 mut260-sal for	ggGGAGTTGGGCCCAcggAAcgCACcgCAcgGTCGCGTCCG
	4 246-284 mut260-sal rev	ggCGGACGCGACcgTGcgGTGcgTTccgTGGGCCCAACTCC
	6 351-379 for	ggCTCGGATTTATGGCCAACAGAAAAAGGCA
	6 351-379 rev	ggTGCCTTTTTCTGTTGGCCATAAATCCGAG
	6 351-379 for mut 359	ggCTCGGATccgTGGCCAACAGAAAAAGGCA
	6 351-379 rev mut 359	ggTGCCTTTTTCTGTTGGCCAcggATCCGAG

Supplementary Table 2: Primary and secondary antibodies used in this work.

**Primaries**				
α-Abd-B 1A2E9	Abdominal B	1:25	mouse	Hybridoma Bank
α-βGal	β galactosidase	1:500	rabbit	MP Biomedicals (8559762)
α-βGal	β galactosidase	1:500	chicken	Abcam (ab13970)
α-βGal	β galactosidase	1:1000	mouse	Promega (Z3781)
α-Ct 2B10	Cut	1:20	mouse	Hybridoma Bank
α-DIG-AP	Digoxigenin alkaline phosphatase conjugate	1:2000	sheep	Roche (11093274910)
α-En 4D9	Engrailed	1:50	mouse	Hybridoma Bank
α-GFP	Green Fluorescent Protein	1:300	rabbit	Invitrogen (A11122)
α-GFP	Green Fluorescent Protein	1:500	chicken	Abcam (ab13970)
α-Sal	Spalt	1:100	rabbit	Our laboratory
α-RFP	Red Fluorescent Protein	1:500	Rat	Chromotek (5F8)
α-Axo49	Axoneme marker	1:500	mouse	Sigma-Aldrich (MABS276)

**Secondaries**				
α-mouse	Alexa Fluor 488	1:200	goat	Invitrogen A-11029
α- mouse	Alexa Fluor 555	1:200	goat	Invitrogen A-21424
α- mouse	Alexa Fluor 647	1:200	goat	Invitrogen A-21236
α- mouse	Biotin	1:100	horse	Vector BA-2000
α-rabbit	Alexa Fluor 488	1:200	goat	Invitrogen A-11034
α- rabbit	Alexa Fluor 555	1:200	goat	Invitrogen A-21429
α- rabbit	Alexa Fluor 647	1:200	goat	Invitrogen A-21245
α-Rat	Alexa Fluor 555	1:200	goat	Invitrogen A-48263
α- chicken	Alexa Fluor 488	1:200	goat	Invitrogen A-32931

Supplementary Table 3: Reporter transgenic lines used in this work.

Reporter transgenic lines	Reporter	Reference
enD	LacZ	19
enH	LacZ	19
enM	LacZ	19
enP	LacZ	19
enX	LacZ	19
enD ds	GFP	This work
enD 0.4	mCherry	This work
*enD 0.4*STAT*	mCherry	This work
*enD 0.4*AbdB*	GFP	This work
sal 2.1	LacZ	This work
sal 2.1	GFP	This work
sal 2.1	mCherry	This work
upd 0.43	LacZ	This work
crb 518	LacZ	37
10XSTAT	GFP	35
STAT92E-GFP BAC	GFP	64

Supplementary Table 4: Male genital lobe measurements in Supplementary Figure 8.

phenotype	date	number	lenght Y	lenght X	Area
OR	28/04/2022	195	315	353	124363
	28/04/2022	192	324	336	108071
	28/04/2022	193	318	330	102502
	28/04/2022	194	290	338	119102
	28/04/2022	191	318	347	122551
	28/04/2022	197	304	314	105502
	28/04/2022	196	300	368	125726
	28/04/2022	189	281	345	109295
	28/04/2022	188	288	354	106407
	28/04/2022	201	297	322	102836
	28/04/2022	198	313	334	112215
	28/04/2022	190	305	356	119756
	02/04/2020	274	312	337	113012
	02/04/2020	280	302	326	96467
	02/04/2020	278	329	324	110633
	02/04/2020	277	320	319	100373
	02/04/2020	276	311	329	107538
	02/04/2020	275	302	325	95721
enD Δ2	02/04/2020	255	275	319	101364
	02/04/2020	254	270	316	94434
	02/04/2020	253	265	326	91637
	02/04/2020	252	270	303	107011
	02/04/2020	235	281	325	100692
	02/04/2020	251	250	317	91464
	02/04/2020	250	293	321	103275
	02/04/2020	249	312	346	110998
	02/04/2020	248	309	346	116129
	02/04/2020	247	278	317	94296
	02/04/2020	246	246	305	93935
	02/04/2020	245	302	306	96388
	02/04/2020	244	252	312	91295
	02/04/2020	243	303	297	91565
	02/04/2020	242	280	307	94506
	02/04/2020	241	280	299	87303

	02/04/2020	240	232	298	75885
	02/04/2020	239	249	304	86489
	02/04/2020	238	264	315	86604
	02/04/2020	237	283	310	89240
	02/04/2020	236	247	296	85490
enD Δ3	02/04/2020	273	252	323	92979
	02/04/2020	272	281	324	103558
	02/04/2020	271	280	330	102402
	02/04/2020	255	316	345	110948
	02/04/2020	270	284	314	94643
	02/04/2020	269	246	332	97559
	02/04/2020	268	288	298	95613
	02/04/2020	267	300	351	117084
	02/04/2020	266	300	327	105783
	02/04/2020	265	255	341	105358
	02/04/2020	264	291	346	97420
	02/04/2020	263	258	323	94809
	02/04/2020	262	232	321	84804
	02/04/2020	261	300	324	112271
	02/04/2020	260	275	343	100697
	02/04/2020	259	288	322	103120
	02/04/2020	258	286	332	103998
	02/04/2020	257	304	320	99094
	02/04/2020	256	258	332	99223
enD Δ2/CyO	05/02/2022	299	308	293	94981
	05/02/2022	307	297	310	99729
	05/02/2022	294	274	278	84580
	05/02/2022	297	302	295	99489
	05/02/2022	298	301	311	107736
	05/02/2022	301	295	276	88690
	05/02/2022	295	278	294	92114
	05/02/2022	300	281	284	81854
	05/02/2022	308	283	300	85143
	05/02/2022	306	277	288	83584
	05/02/2022	304	273	302	91811
	05/02/2022	309	330	288	99774
	05/02/2022	303	298	279	89968

	05/02/2022	305	319	289	103233
	05/02/2022	296	271	293	84614
	05/02/2022	293	260	292	85427
	05/02/2022	292	312	297	94267
	02/04/2020	281	294	305	93006
	02/04/2020	282	277	310	96081
	02/04/2020	283	285	303	88976
	02/04/2020	284	295	308	88826
	02/04/2020	285	297	293	87107
	02/04/2020	286	297	329	105678
	02/04/2020	287	298	313	102351
	02/04/2020	288	296	304	96754
	02/04/2020	289	306	298	96710
	02/04/2020	290	282	308	92841
	02/04/2020	291	275	290	86850

Supplementary Table 5. Details of pairwise *post hoc* Tukey-Kramer test.

		95% confidence interval		
Comparisons	Mean difference	lower	upper	p-value
enDΔ2/Cyo-enDΔ2	-1827,119	-8046,97	4392,733	0,867
enDΔ3-enDΔ2	6362,464	-459,576	13184,503	0,076
OR-enDΔ2	15353,095	8432,283	22273,908	>0,001
enDΔ3-enDΔ2/Cyo	8189,583	1785,399	14593,767	0,007
OR-enDΔ2/Cyo	17180,214	10670,91	23689,515	>0.001
OR-enDΔ3	8990,632	1903,696	16077,567	0,007

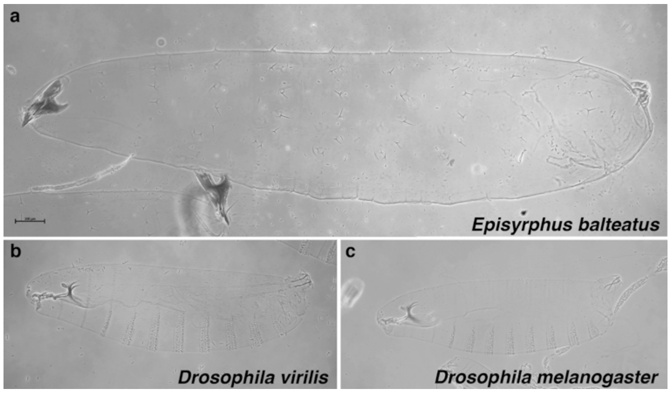

Supplementary Figure 1. Cuticles of early hatched dipteran larvae.
(a) *Episyrphus balteatus*, (b) *Drosophila virilis*, (c) *Drosophila melanogaster*. Scale bar: 100 µm applies to all panels.

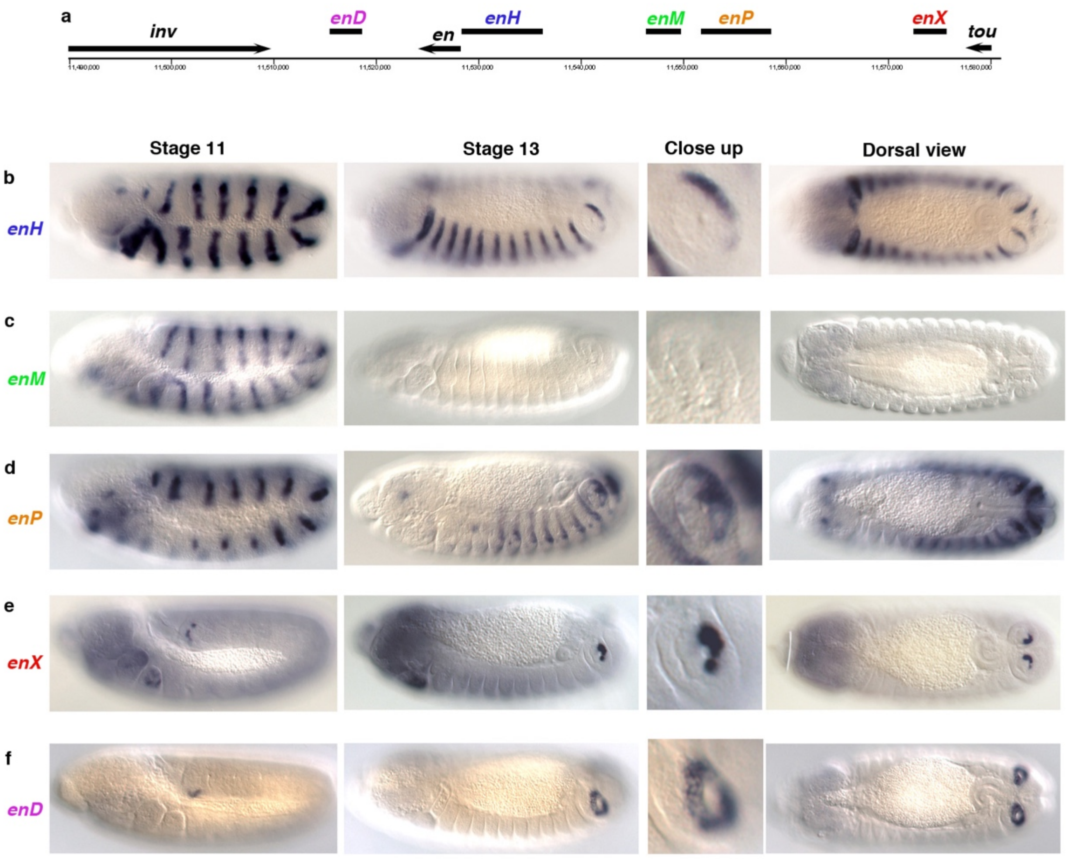

Supplementary Figure 2. Embryonic expression mediated by different *en* CRE.
(a) Schematic representation of the *en* locus showing the localization of selected CREs (boxes) and transcription units (arrows). (b-f) RNA *in situ* showing *lacZ* expression driven by five different *en* CREs at st11 and st13. Close ups show expression in dorsal A8. Panels on the right column show st13 dorsal views and on the left and centre, lateral views. (b) *enH* drives posterior compartment expression at st11. Expression is maintained at st13 except in ventral A8. (c) *enM* is expressed at st11 but not at st13. (d) *enP* is expressed at st11 and st13 in the posterior compartment of the abdominal segments. (e) *enX* expression is restricted to small cell groups that we believe to be spiracle sensory elements due to their position and shape. (f) *enD* expression becomes specifically activated at st11 in dorsal anterior A8 cells and expands to form a circumference around the spiracle opening at st13.

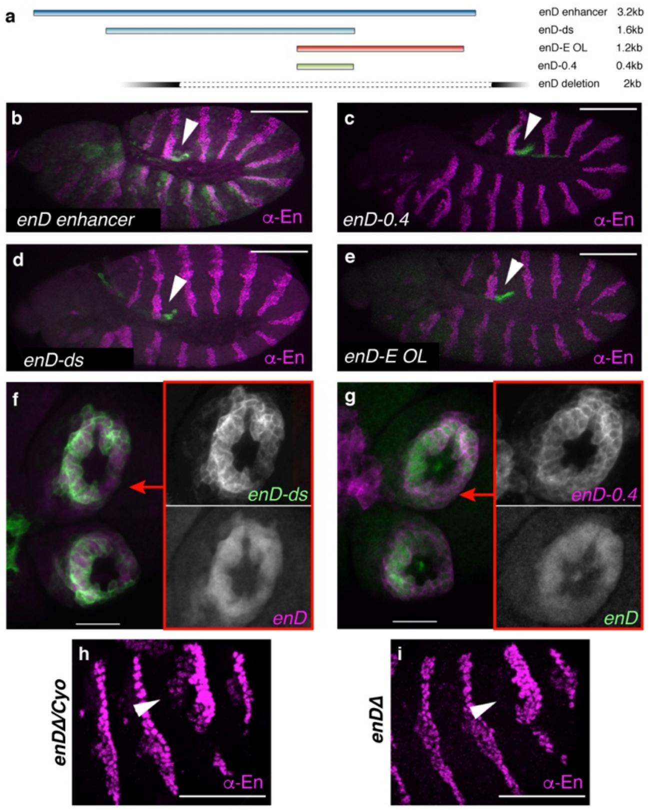

Supplementary Figure 3. Dissection of the *enD* posterior spiracle CRE.
(a) Scheme of the fragments studied to localise the posterior spiracle CRE and relative location of the *enDΔ* CRISPR-Cas9 2kb deletion. (b-e) St11 embryos expressing either *lacZ*, GFP or mCherry (green) double stained with anti-En (magenta). (b) anti-ßGal in *enD-lacZ*. (c) anti-mCherry in *enD0.4-mCherry*. (d) anti-GFP in *enD-ds-PH-GFP* or (e) *enD-E overlapping-PH-GFP*. (f-g) *enD-lacZ* combined with *enD-ds-PH-GFP* (f) or with *enD0.4-mCherry* (g) double stained with anti-βGal and either anti-GFP or anti-mCherry to show their almost identical expression in the spiracles. (h-i) En protein expression (magenta) in st13 embryos heterozygous (h) or homozygous (i) for the *enDΔ* deletion. Note in (i) the absence of anterior En expression from A8a, showing the enhancer is not redundant. The *enD-lacZ* fragment has low general expression in the trunk that is absent in smaller constructs. Some *enD* reporter lines show ectopic expression in the amnioserosa which is not an *engrailed* expression feature. Arrowheads point to the anterior A8 dorsal region where En becomes activated. Scale bar in (b-e) and (h-i): 50 μm; in (f-g): 20 μm.

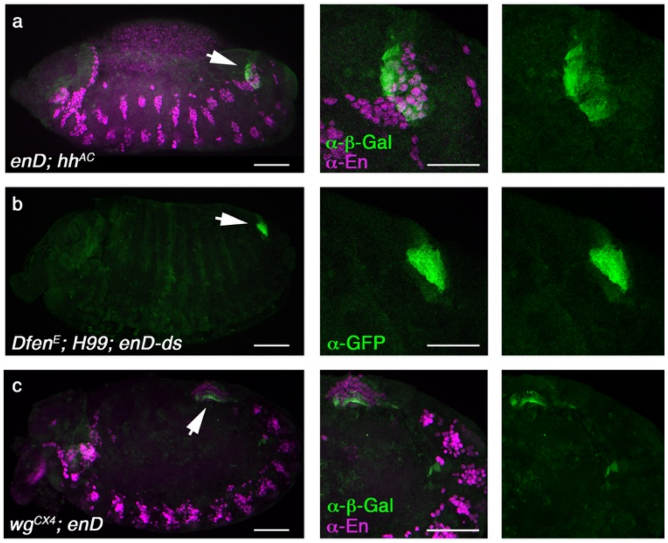

Supplementary Figure 4. Expression of *enD-lacZ* in mutants for the segmentation gene network.

(a-c) Expression of En (magenta) and βGal or GFP (green) in st14 *enD-lacZ* embryos homozygous mutant for hh^{AC} (a), *Df(2)enE; Df (3L)H99* (b) or wg^{CX4} (c). Right panels show single channel images in a close up of the spiracle region. Despite the posterior spiracle's abnormal shape, the *enD* enhancer maintains its expression in A8 (arrows). Scale bar: 50 μm.

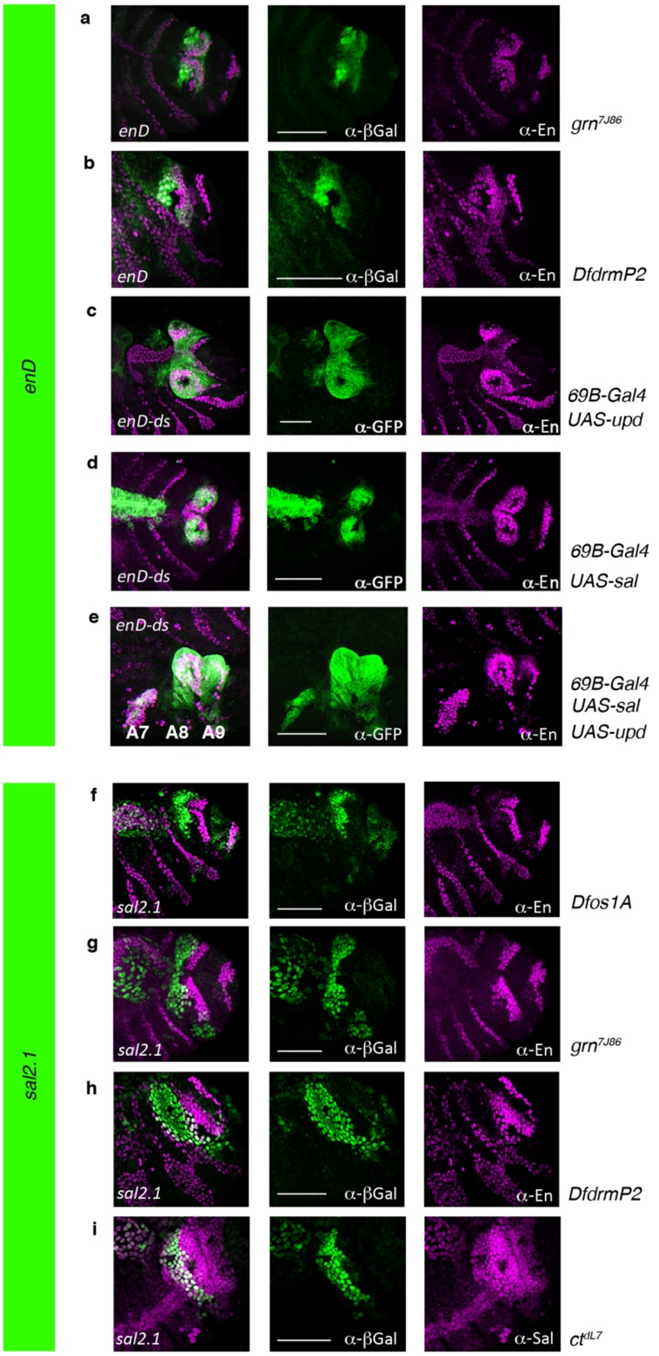

Supplementary Figure 5. Expression of *enD* and *sal2.1* in mutants for the posterior spiracle gene network. (a-b) Expression of *enD-lacZ* in *grn*^{7J86} (a) or in *Df(2L)drmP2* mutants deleting *drm*, *sob* and *odd* (b). (c-e) *enD-GFP* expression in embryos where the *69B-Gal4* line drives ectodermal ectopic expression of *UAS-upd* (c), *UAS-sal* (d) or both *UAS-upd* and *UAS-sal* (e). (f-i) Expression of *sal2.1-lacZ* in *Df(1)os1A* deleting all Upd ligands (f), in *grn*^{7J86} mutants (g), in *Df(2L)drmP2* (h) or in *ct*^{dl7} mutants (i). All embryos are double stained with anti-ßGal or anti-GFP (green) and in magenta with anti-En (a-h) or anti-Sal (i). Scale bar: 50 μm.

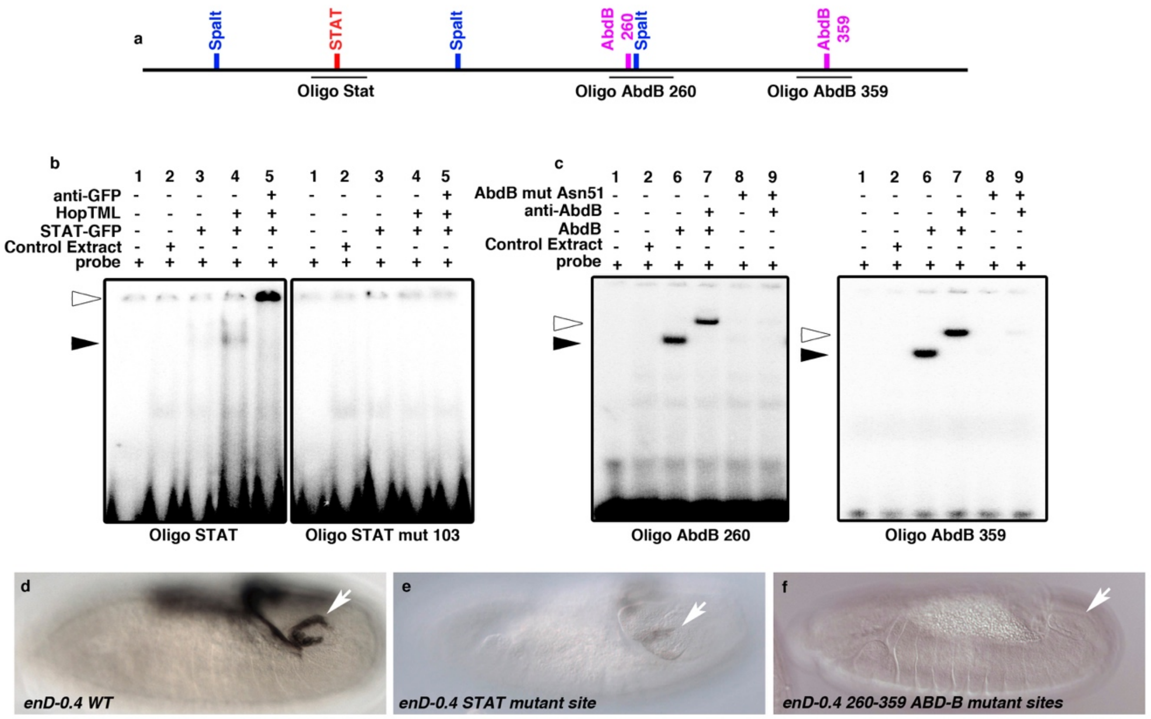

Supplementary Figure 6. Direct binding of STAT and Abd-B to *enD0.4*.
(a) Schematic representation of *enD0.4* showing the location of some putative STAT, Sal and Abd-B binding sites and the oligos used in panels b and c for EMSA. (b) EMSA showing activated STAT-GFP (by co-expression with the JAK Hop Tml kinase) binding to an oligo containing a wild type 4nSTAT site at position 103 (lane 4, left gel, black arrowhead) and its super retardation after adding an anti-GFP antibody (lane 5, left gel, white arrowhead); but not when the predicted STAT binding site is mutated (lane 4 and 5, right gel). (c) EMSA showing full-length Abd-B binding to an oligo containing the predicted Abd-B binding site at position 260 (lane 6 black arrowhead, left gel) or an oligo containing site 359 (lane 6, right gel). Lane 7 in both gels shows Abd-B binding super retardation with the anti-Abd-B antibody (white arrowhead). An AbdBAsn51 mutant protein affecting the homeodomain is unable to bind the same oligos (lanes 8 and 9). (d-f) Embryos at st13 showing the expression driven by the wild type *enD0.4-GFP* reporter (d), driven by the *enD0.4stat mut-GFP* reporter (e) or the expression driven by the *enD0.4 260-359 mut-GFP* reporter with the 260 and 359 Abd-B putative sites mutated (f). White arrows in d-f point at the stigmatophore. Note that some *enD0.4 wt* transgenic lines also have ectopic expression in the amnioserosa membrane.

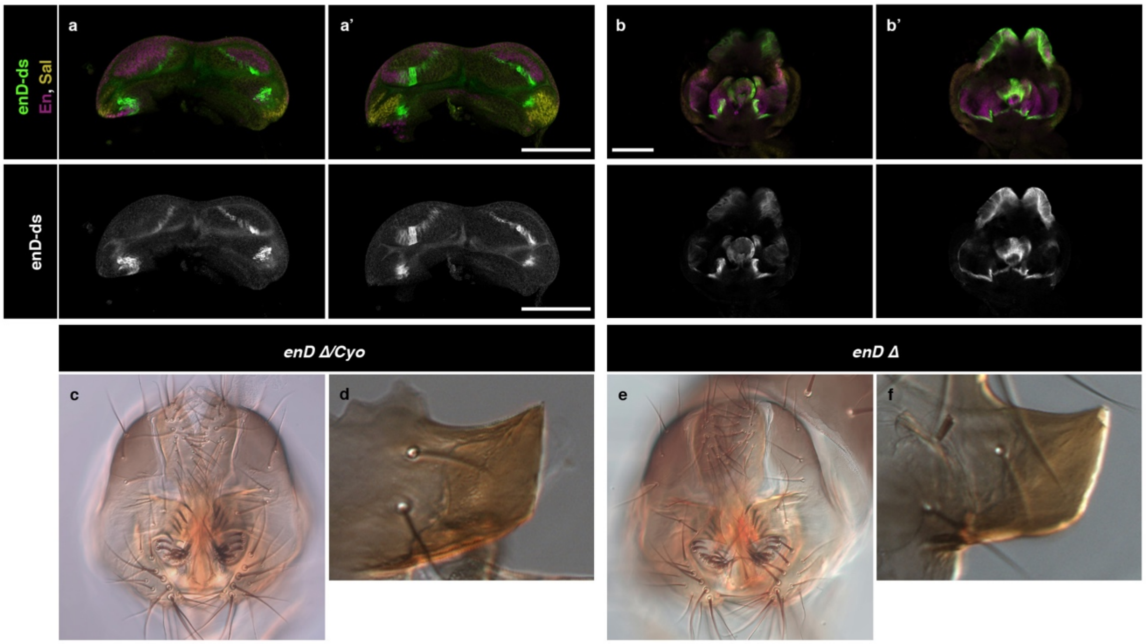

Supplementary Figure 7. Expression of *enD* in genital discs and morphology of adult genitalia in *enD*Δ mutant males.

(a-b) Expression of *enDds-GFP* reporter (green or white), Engrailed (magenta) and Spalt (yellow) proteins in the male genital discs of a third instar larva (a, surface and a' medial focal plane) or of a pupa 48h after puparium formation (b, surface and b' medial focal plane). (c-d) External genitalia in control heterozygous males and in (e-f) *enDΔ3* homozygous males. (d, f) show dissected posterior lobes. Scale bar: 20µm.

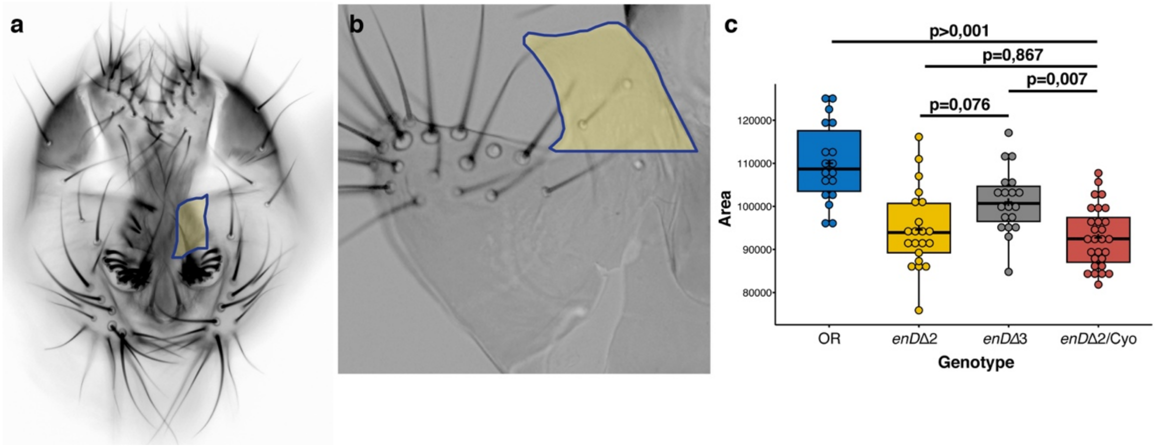

Supplementary Figure 8. Posterior lobe variability in control and *enD* homozygous mutants.

(a) Adult wild type male genitalia. (b) Highlighted posterior lobe area in a dissected male genitalia. (c) Boxplot showing the posterior lobe area for each genotype measured in pixels (Oregon-R, n=18, or *enDΔ2*/CyO, n=28, males compared to *enDΔ2*, n=21, and *enDΔ*3, n=19, homozygous males). A two-sided *post hoc* Tukey HDS test was considered to compare the posterior lobe area between genotypes. Boxplot for the area measurement for each phenotype. Boxplots indicate the median (bold line near the centre), mean (bold cross near the centre), the first and third quartile (the box), the extreme values whose distance from the box is at most 1.5 times the inter quartile range (whiskers), and remaining outliers (dots outside the whiskers). All measurements are shown as dots. Lines above the boxplots indicate pairwise comparisons. p-values according to pairwise *post hoc* Tukey-Kramer test are shown (also Sup. Table 5). Source Data are provided as a Source Data file.

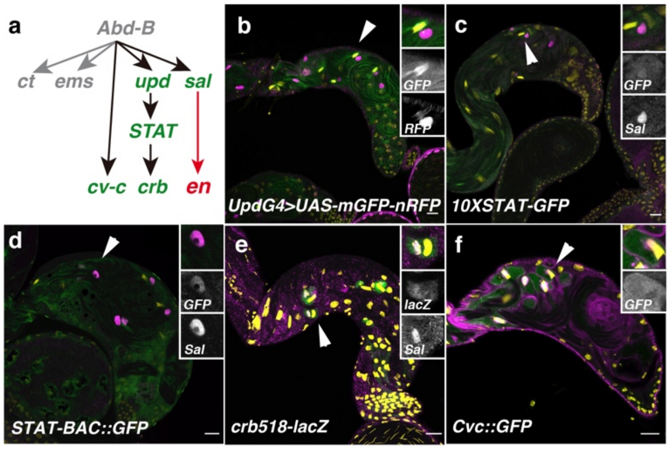

Supplementary Figure 9. Expression of posterior spiracle genes in the testis.
(a) Scheme of the posterior spiracle gene network highlighting in green or red the genes that are also expressed in the HCCs and in grey those that are not. (b-f) *Drosophila melanogaster* testis terminal region stained for various posterior spiracle genes. (b) *upd-Gal4* line driving expression of membrane GFP (green) and nuclear RFP (magenta). (c) *10XSTAT-GFP* reporter (green) of JAK/STAT pathway activation. (d) STAT92E::GFP (green). (e) *crb518-lacZ* (green) posterior spiracle enhancer expression in the HCCs. (f) Endogenous Cv-c protein fused to GFP can be detected in the cytoplasm of HCCs (green). (c-e) Testes stained with anti-Sal to label the HCC nuclei (magenta). (f) is also stained with Rhodamine phalloidin (magenta). DAPI DNA staining in yellow. Scale bar: 20µm.

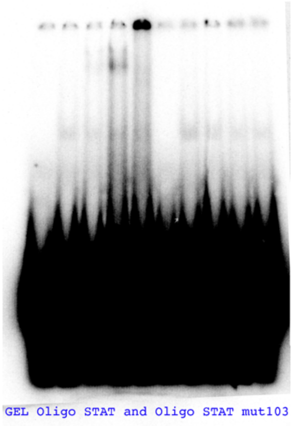

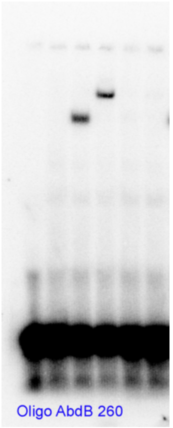

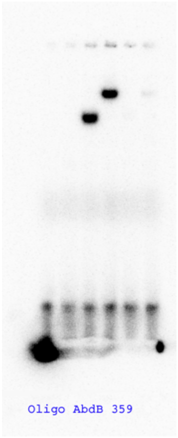

Source data for Supplementary Figure 6.
The uncropped gels used in Supplementary Figure 6 are shown keeping the same layout used in the figure.
