# Supplementary material for: Interlocking of co-opted developmental gene networks in Drosophila and the evolution of pre-adaptive novelty
Source: Nat Commun. 2023 Sep 15;14:5730. doi: 10.1038/s41467-023-41414-3 (PMC10504328; doi:10.1038/s41467-023-41414-3)
Supplement: Supplementary file 3 — Reporting Summary [file 41467_2023_41414_MOESM3_ESM.pdf]

nature portfolio

Corresponding author(s): James C-G Hombria

Last updated by author(s): Aug 11, 2023

Reporting Summary

Nature Portfolio wishes to improve the reproducibility of the work that we publish. This form provides structure for consistency and transparency in reporting. For further information on Nature Portfolio policies, see our Editorial Policies and the Editorial Policy Checklist.

Statistics

For all statistical analyses, confirm that the following items are present in the figure legend, table legend, main text, or Methods section.

n/a	Confirmed	
☐	☒	The exact sample size (*n*) for each experimental group/condition, given as a discrete number and unit of measurement
☐	☒	A statement on whether measurements were taken from distinct samples or whether the same sample was measured repeatedly
☐	☒	The statistical test(s) used AND whether they are one- or two-sided<br>*Only common tests should be described solely by name; describe more complex techniques in the Methods section.*
☒	☐	A description of all covariates tested
☒	☐	A description of any assumptions or corrections, such as tests of normality and adjustment for multiple comparisons
☐	☒	A full description of the statistical parameters including central tendency (e.g. means) or other basic estimates (e.g. regression coefficient) AND variation (e.g. standard deviation) or associated estimates of uncertainty (e.g. confidence intervals)
☐	☒	For null hypothesis testing, the test statistic (e.g. *F*, *t*, *r*) with confidence intervals, effect sizes, degrees of freedom and *P* value noted<br>*Give P values as exact values whenever suitable.*
☒	☐	For Bayesian analysis, information on the choice of priors and Markov chain Monte Carlo settings
☒	☐	For hierarchical and complex designs, identification of the appropriate level for tests and full reporting of outcomes
☒	☐	Estimates of effect sizes (e.g. Cohen's *d*, Pearson's *r*), indicating how they were calculated

Our web collection on statistics for biologists contains articles on many of the points above.

Software and code

Policy information about availability of computer code

Data collection	Leica Stellaris and Leica SPE software using the company's provided software
Data analysis	Images were processed using FIJI ImageJ2 (version 2.9.0/1.54f), Imaris x64 (version 8.0.2) and Adobe Photoshop CS5 (version 12.0 x64) programs. Data were analyzed with Microsoft Excel (version 16.16.27) and GraphPad Prism 8 (version 8.4.3 (471)).

For manuscripts utilizing custom algorithms or software that are central to the research but not yet described in published literature, software must be made available to editors and reviewers. We strongly encourage code deposition in a community repository (e.g. GitHub). See the Nature Portfolio guidelines for submitting code & software for further information.

Data

Policy information about availability of data

All manuscripts must include a data availability statement. This statement should provide the following information, where applicable:

- Accession codes, unique identifiers, or web links for publicly available datasets
- A description of any restrictions on data availability
- For clinical datasets or third party data, please ensure that the statement adheres to our policy

All data generated in this study are available within the Article and Supplementary Files. There are no data restrictions. Fly stocks can be freely obtained upon request.

Research involving human participants, their data, or biological material

Policy information about studies with human participants or human data. See also policy information about sex, gender (identity/presentation), and sexual orientation and race, ethnicity and racism.

Reporting on sex and gender	N/A
Reporting on race, ethnicity, or other socially relevant groupings	N/A
Population characteristics	N/A
Recruitment	N/A
Ethics oversight	N/A

Note that full information on the approval of the study protocol must also be provided in the manuscript.

Field-specific reporting

Please select the one below that is the best fit for your research. If you are not sure, read the appropriate sections before making your selection.

- [x] Life sciences
- [ ] Behavioural & social sciences
- [ ] Ecological, evolutionary & environmental sciences

For a reference copy of the document with all sections, see nature.com/documents/nr-reporting-summary-flat.pdf

Life sciences study design

All studies must disclose on these points even when the disclosure is negative.

Sample size	More than 20 animals of the relevant genotypes and temporal stage were studied in each case. Phenotypes were very homogeneous so no sample size calculations needed to be performed.
Data exclusions	No data were excluded
Replication	Three replicates were studied, with all replicates being succesful
Randomization	These is not relevant, we have to select the correct genotype to study
Blinding	All genotypes were unambiguously labelled with genetic markers that allowed obtaining internal controls in every experiment. Due to the homogeneous phenotypes observed, blinding was not found to be relevant for the data analyses presented. Negative and positive controls were sufficient.

Reporting for specific materials, systems and methods

We require information from authors about some types of materials, experimental systems and methods used in many studies. Here, indicate whether each material, system or method listed is relevant to your study. If you are not sure if a list item applies to your research, read the appropriate section before selecting a response.

Materials & experimental systems

n/a	Involved in the study	
[ ]	[x]	Antibodies
[ ]	[x]	Eukaryotic cell lines
[x]	[ ]	Palaeontology and archaeology
[ ]	[x]	Animals and other organisms
[x]	[ ]	Clinical data
[x]	[ ]	Dual use research of concern
[x]	[ ]	Plants

Methods

n/a	Involved in the study	
[x]	[ ]	ChIP-seq
[x]	[ ]	Flow cytometry
[x]	[ ]	MRI-based neuroimaging

Antibodies

Antibodies used	Primary antibodies used: mouse α-Ct 2B10 1:20 (DSHB), mouse α-Abd-B 1A2E9 1:25 (DSHB, mouse anti-en 1:50 (DSHB, 4D9), rabbit α-sal 82, rat anti-RFP 1:500 (Chromotek, 5F8), mouse anti-Axo49 1:500 (Sigma-Aldrich, MABS276), mouse anti-βgal 1:1.000 (Promega, Z378A), chicken anti-βgal 1:500 (Abcam 9361), rabbit anti-βgal 1:1.000 (Promega, Z378A) rabbit anti-GFP 1:300 (Invitrogen, A11122), chicken anti-GFP 1:500 (Abcam, ab13970).

	Secondary antibodies: α-mouse Alexa Fluor 488 1:200 goat Invitrogen A-11029; α-mouse Alexa Fluor 555 1:200 goat Invitrogen A-21424; α-mouse Alexa Fluor 647 1:200 goat Invitrogen A-21236; α-mouse Biotin 1:100 horse Vector BA-2000; α-rabbit Alexa Fluor 488 1:200 goat Invitrogen A-11034; α-rabbit Alexa Fluor 555 1:200 goat Invitrogen A-21429; α-rabbit Alexa Fluor 647 1:200 goat Invitrogen A-21245; α-rat Alexa Fluor 555 1:200 goat Invitrogen A-48263; α-chicken Alexa Fluor 488 1:200 goat Invitrogen A-32931. see suplementary table 2 in manuscript. Filamentous Actin was stained with Rhodamine phalloidin (Molecular Probes, R415). For RNA in situ hybridization α-DIG-alcaline phosphatase conjugated was used (1:2000) (Roche)
Validation	Previously tested in other authors' publications

Eukaryotic cell lines

Policy information about cell lines and Sex and Gender in Research

Cell line source(s)	S2R+ cells (DGRC Stock 150; https://dgrc.bio.indiana.edu//stock/150; RRID:CVCL_Z831)
Authentication	modENCODE line authenticated
Mycoplasma contamination	lines not tested for Mycoplasma contamination
Commonly misidentified lines (See ICLAC register)	N/A

Animals and other research organisms

Policy information about studies involving animals; ARRIVE guidelines recommended for reporting animal research, and Sex and Gender in Research

Laboratory animals	Drosophila melanogaster, Drosophila virilis and Episyrphus balteatus embryos of all stages from 0-17 hours, late L3 larvae and five day old males.
Wild animals	This study did not use wild animals
Reporting on sex	For embryonic analyses both sexes were analysed. For male genitalia and testis analyses only males were studied as females were fully fertile.
Field-collected samples	No samples were collected from the field
Ethics oversight	The Spanish Ministry of Agriculture gave permission for working with transgenic Drosophila flies.

Note that full information on the approval of the study protocol must also be provided in the manuscript.
